# Diagnostic Overlap Between Uremia and Severe Hyperkalemia in Chronic Kidney Disease: Emphasizing Laboratory-Guided Urgency Despite Absent EKG Changes

**DOI:** 10.7759/cureus.107246

**Published:** 2026-04-17

**Authors:** Muhammad Hasnain Tahir, Fatima Tahir, Muhammad Mashhood Tahir, Abdullah Imran, Samina Asghar

**Affiliations:** 1 Cardiovascular Medicine, Stanford University Medical Center, San Francisco, USA; 2 Internal Medicine, Faisalabad Medical University, Faisalabad, PAK; 3 Community Dentistry, Avicenna Medical College, Lahore, PAK; 4 Obstetrics and Gynaecology, Gujranwala Teaching Hospital, Gujranwala, PAK

**Keywords:** acute-on-chronic kidney disease, hemodialysis, hyperkalemia, nausea, serum potassium, urea reduction, uremic gastritis

## Abstract

Hyperkalemia is a potentially life-threatening electrolyte disturbance often associated with characteristic electrocardiographic (EKG) changes and neuromuscular manifestations. However, clinical presentation does not always correlate with biochemical severity.

We present the case of a 53-year-old female with chronic kidney disease (CKD) who developed acute-on-chronic kidney injury with severe hyperkalemia (serum potassium 8.7 mmol/L) and elevated urea (94 mg/dL), yet without EKG abnormalities or overt symptoms. The patient reported persistent nausea for 24 hours without abdominal pain or hemodynamic instability. Urgent hemodialysis was initiated due to critically elevated potassium. Post-dialysis, potassium normalized to 5.6 mmol/L, urea improved, and nausea resolved within one to two hours, suggesting a possible association between hyperkalemia and the presenting symptoms.

This case highlights the diagnostic overlap between uremia and severe hyperkalemia and underscores the importance of laboratory-driven urgency in CKD patients, as significant hyperkalemia may be present despite minimal symptoms and normal EKG findings.

## Introduction

Hyperkalemia is a common and potentially fatal electrolyte disturbance, particularly among patients with chronic kidney disease (CKD) [1,2]. Severe hyperkalemia (serum potassium ≥7.0 mmol/L) can precipitate life-threatening arrhythmias and requires prompt intervention [2,3]. Classical electrocardiographic (EKG) manifestations include peaked T waves, QRS widening, and conduction abnormalities; however, the correlation between serum potassium levels and EKG changes is inconsistent, especially in patients with chronic elevations, where physiological adaptation may occur [3,4].

Patients with CKD often present with nonspecific gastrointestinal symptoms such as nausea, vomiting, and anorexia, frequently attributed to uremia. These overlapping features can obscure recognition of critical electrolyte disturbances, potentially delaying treatment [4,5].

This case highlights that severe hyperkalemia may remain clinically silent and emphasizes the importance of laboratory-guided intervention in CKD patients. We report a case of severe hyperkalemia in acute-on-chronic kidney injury where laboratory findings, rather than symptoms or EKG changes, guided urgent intervention, illustrating the importance of vigilance in similar clinical scenarios.

## Case presentation

A 53-year-old female with a known history of stage 3 CKD (baseline creatinine 1.6 mg/dL and urea 33 mg/dL) presented with a 24-hour history of persistent nausea. She denied vomiting, abdominal pain, palpitations, chest pain, or generalized weakness. On admission, she was hemodynamically stable, with a blood pressure of 130/78 mmHg, heart rate of 82 beats per minute, respiratory rate of 16 breaths per minute, oxygen saturation of 98% on room air, and she was afebrile.

The medication history revealed no use of angiotensin-converting enzyme (ACE) inhibitors, angiotensin receptor blockers (ARBs), potassium-sparing diuretics, or nephrotoxic drugs. Precipitating factors for acute kidney injury (AKI) included recent reduced oral intake and relative dehydration. The patient was clinically euvolemic with adequate urine output.

Initial laboratory investigations revealed severe hyperkalemia with a serum potassium level of 8.7 mmol/L. Blood urea nitrogen was elevated at 94 mg/dL, and serum creatinine was 4.6 mg/dL, consistent with acute-on-chronic kidney injury. Serum sodium, calcium, and hemoglobin levels were within acceptable limits, while bicarbonate was mildly reduced at 20 mmol/L. Electrocardiography demonstrated a normal sinus rhythm without evidence of peaked T waves, QRS widening, or conduction abnormalities. A summary of laboratory parameters before and after hemodialysis is presented in Table 1.

**Table 1:** Laboratory parameters before and after a single hemodialysis session in a patient with acute-on-chronic kidney disease. The table shows serum potassium, urea, creatinine, sodium, calcium, bicarbonate, and hemoglobin levels measured immediately prior to and after dialysis.

Parameter	Reference Range	Before Dialysis	After Dialysis	Notes
Serum potassium (K⁺)	3.5-5.5 mmol/L	8.7	5.6	Severe hyperkalemia; normalized post-dialysis
Serum urea (blood urea nitrogen)	15-45 mg/dL	94	42	Elevated due to acute-on-chronic kidney injury
Serum creatinine	0.6-1.2 mg/dL	4.6	2.1	Elevated, baseline 1.6 mg/dL
Sodium (Na⁺)	135-145 mmol/L	138	137	Within normal range
Calcium (Ca²⁺)	8.5-10.5 mg/dL	9	8.8	Within normal range
Bicarbonate (HCO₃⁻)	22-28 mmol/L	20	22	Corrected with dialysis
Hemoglobin	12-16 g/dL	11.2	11.2	Stable

Management

Temporizing measures (IV calcium, insulin/glucose, beta-agonists) were considered, but urgent hemodialysis was prioritized due to critically elevated potassium. The patient tolerated the procedure well, and no arrhythmias were observed during treatment. Post-dialysis laboratory evaluation showed a reduction in serum potassium to 5.6 mmol/L, along with improvement in urea to 42 mg/dL and creatinine to 2.1 mg/dL, while bicarbonate levels improved to 22 mmol/L. Notably, the patient’s nausea resolved completely within one to two hours following dialysis, suggesting a temporal association with correction of hyperkalemia, although causality cannot be definitively established.

## Discussion

This case highlights the diagnostic challenge posed by overlapping manifestations of uremia and severe hyperkalemia in patients with CKD, demonstrating that biochemical severity may not correlate with classical clinical or EKG findings. Patients with CKD often present with nonspecific gastrointestinal symptoms, such as nausea, which can be attributed to either uremia or electrolyte disturbances. Hyperkalemia is a frequent complication of CKD due to impaired renal potassium excretion and adaptive physiological mechanisms.

In this patient, the rapid resolution of nausea following hemodialysis suggests that hyperkalemia may have been a significant contributor to the presenting symptoms, although mild uremia or metabolic factors cannot be entirely excluded. Chronic elevations in potassium may be partially tolerated through cellular and renal adaptations, particularly when the rise occurs gradually, as seen in acute-on-chronic settings [1,2].

Although severe hyperkalemia is traditionally considered a medical emergency due to its potential to induce life-threatening arrhythmias, several studies have demonstrated a poor correlation between serum potassium levels and EKG abnormalities, especially in patients with advanced kidney disease [3]. This EKG-potassium discordance can be explained by chronic adaptation to elevated potassium levels, including myocardial cellular adaptation. Consequently, reliance on EKG findings alone may lead to underestimation of risk. While temporizing measures such as intravenous calcium, insulin with glucose, and beta-agonists were considered, urgent hemodialysis was prioritized due to the life-threatening risk associated with severe hyperkalemia.

At the same time, CKD is associated with the accumulation of uremic toxins, which can produce nonspecific gastrointestinal symptoms such as nausea, anorexia, and dyspepsia [4,5]. These symptoms overlap significantly with those seen in metabolic and electrolyte disturbances, complicating clinical assessment.

Medication history revealed no use of ACE inhibitors, ARBs, or potassium-sparing diuretics, and precipitating factors for AKI included recent reduced oral intake and relative dehydration; the patient was clinically euvolemic with adequate urine output. Alternative contributors, including mild uremia or metabolic acidosis, were considered but were unlikely to fully explain the rapid symptom resolution.

However, the rapid resolution of symptoms within one to two hours following a single hemodialysis session supports a possible association with correction of hyperkalemia, while recognizing that uremic symptoms may also improve with dialysis and cannot be completely excluded.

This case underscores the importance of laboratory-driven clinical decision-making in CKD patients presenting with nonspecific symptoms. It reinforces that severe hyperkalemia may remain clinically silent or minimally symptomatic despite posing a significant risk. Early recognition and prompt intervention, guided by laboratory findings rather than clinical presentation alone, are essential to prevent adverse outcomes. Clinicians should maintain a low threshold for urgent evaluation and management of electrolyte abnormalities in patients with acute-on-chronic kidney dysfunction [5-7]. Documenting clinical context enhances the educational value of such cases.

## Conclusions

Severe hyperkalemia may present without classical clinical or EKG findings in patients with CKD, creating a potential diagnostic challenge due to overlap with uremic symptoms. This case highlights that reliance on symptoms or EKG findings alone may underestimate the severity of underlying electrolyte disturbances. Instead, laboratory abnormalities should prompt timely and decisive intervention, as delayed recognition of severe hyperkalemia can have life-threatening consequences. Clinicians should maintain a high index of suspicion and prioritize biochemical data when evaluating CKD patients with nonspecific symptoms to ensure prompt and appropriate management.
